# Supplementary material for: Multiple roles of H2A.Z in regulating promoter chromatin architecture in human cells
Source: Nat Commun. 2021 May 5;12:2524. doi: 10.1038/s41467-021-22688-x (PMC8100287; doi:10.1038/s41467-021-22688-x)
Supplement: Supplementary file 1 — Supplementary Information [file 41467_2021_22688_MOESM1_ESM.pdf]

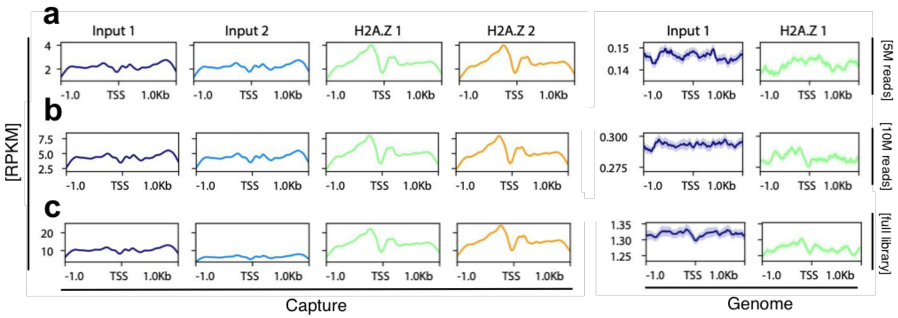

d

Library	Min.	1st Quartile	Median	Mean	3rd Quartile	Max.	Fold-change (mean)
H2A.Z capture 5M	0	0	0.03	2.58	1.69	65.2	18.4
H2A.Z genome 5M	0	0	0.09	0.14	0.17	8.59	
H2A.Z capture 10M	0	0	0.03	5.14	3.38	129	18.4
H2A.Z genome 10M	0	0.09	0.18	0.28	0.35	16.9	
H2A.Z capture full	0	0	0.09	14.8	9.71	3.74	11.7
H2A.Z genome 45M	0	0.51	0.94	1.27	1.5	68.8	

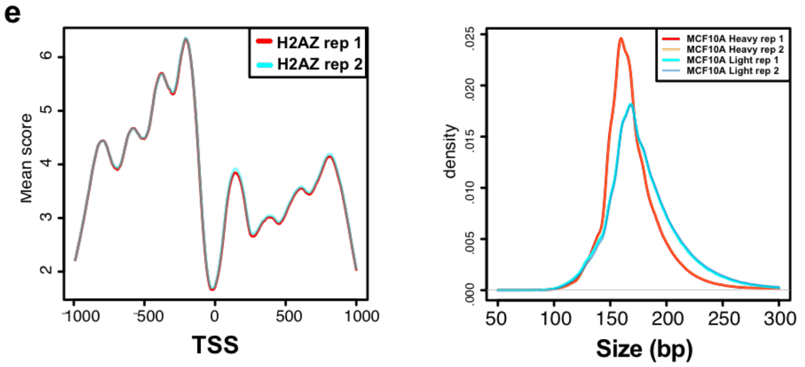

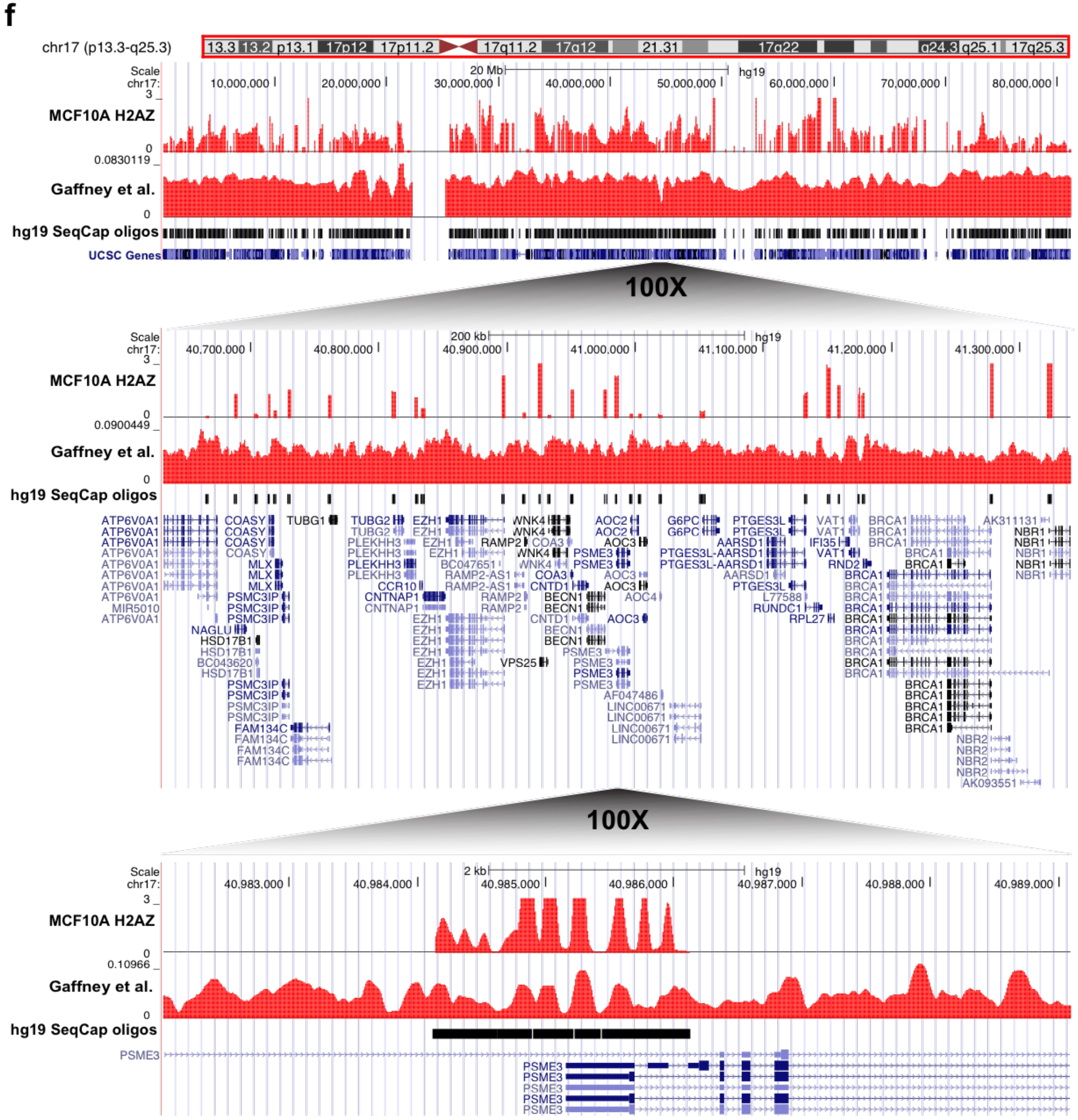

Supplementary Fig. 1. Specific enrichment of H2A.Z at promoters using mTSS-seq. Average coverage profiles (corrected for library size and input subtracted) across the promoter regions of genes covered by our capture array, showing mean coverage (+/- standard error). The profiles for three different sequencing depths are plotted for the capture and genome-wide libraries. In order to demonstrate the performance of the capture approach, all libraries were subsampled to 5M (**a**) and 10M (**b**) PE reads. **c**, Fully sequenced capture libraries were included as well as genome wide ChIP libraries subsampled to 45M

PE reads. The coverage profiles for the capture libraries are highly homogenous. The coverage profiles for the genome libraries show higher variability across all sizes and display higher variability in the obtained average coverage profiles. **d**, calculated mean coverages in 10bp windows across all defined promoter regions for each of the libraries. At 5M reads there is an 18.4-fold mean enrichment, which remains near constant at 10M reads (18.3-fold) indicating a linear increase in coverage in both library types. In the fully sequenced libraries, we observed a mean coverage of 14.8 in the capture library versus 1.27 in the conventional library (a 11.65-fold enrichment). However, the capture libraries for H2A.Z ChIP were sequenced to approximately 27M and 30M PE reads versus subsampled 45M PE reads for the conventional H2A.Z ChIP library, indicating a significantly improved TSS coverage in the capture libraries and thus demonstrating the advantage of using this technology for studying specific regions of the genome at very high resolution. Only at the resolution achieved with the use of the capture technology were we able to distinguish the patterns of nucleosome positioning that underly this study. These promoter patterns were not discernible in the genome-wide libraries even when using the data from the fully sequenced libraries which yielded 277M reads, and thus are virtually at saturation (data not shown). **e**, Average plot of sequence-captured MCF10A H2AZ ChIP-seq data (rep1 and rep2) +/- 1000bp surrounding the TSS and size distribution of fragments in heavy and light MNase digests in MCF10A cells. **f**, Schematic demonstrating targeted enrichment with hg19 SeqCap oligos (mTSS-seq). MCF10A H2AZ data for chromosome 17 shown in the UCSC genome browser (genome.ucsc.edu), with multiple magnifications down to a single TSS, PSME3, compared to a previously published nucleosome mapping data set[1].

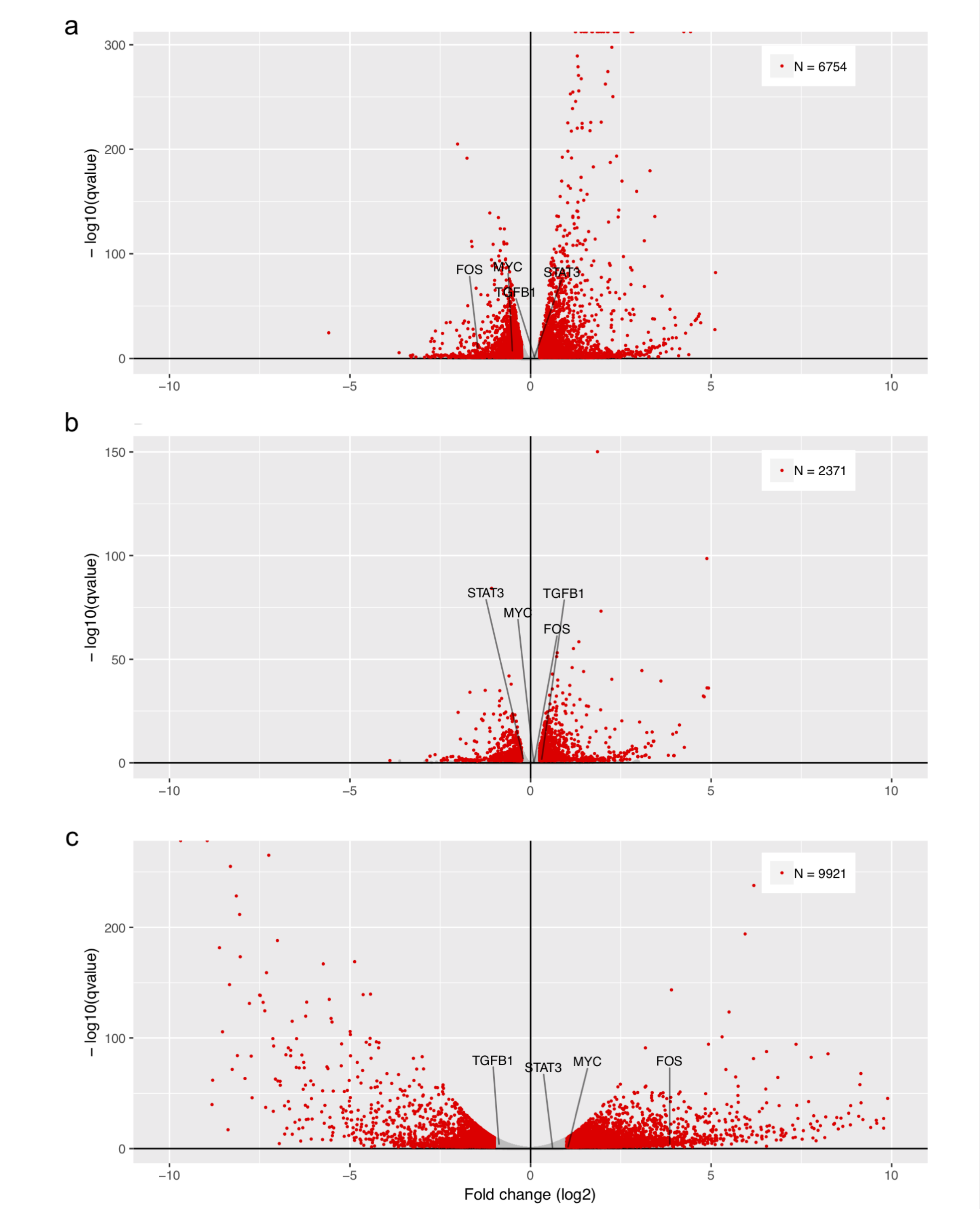

Supplementary Fig. 2. Differential expressed genes. a, Differentially expressed genes between MCF-10A and MCF-10A + TGF-β cells. Log_2 fold-changes in gene expression are plotted against the inverted log_{10}-transformed q-values[2]. **b**, Differentially expressed genes between MCF-10A and shH2A.Z MCF-10A cells. **c**, Differentially expressed genes between MCF-10A and MCF-10CA1a cells.

A

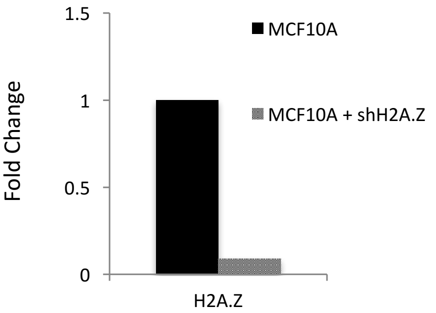

B

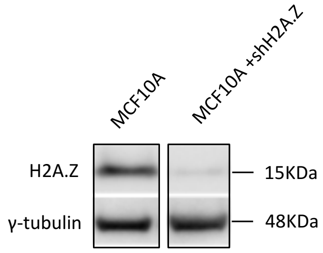

Supplementary Fig. 3. The knockdown of H2A.Z expression in MCF-10A cells. MCF10A cells were transduced with or without pLVTHM shH2A.Z. **a,** Gene expression of H2A.Z was determined by qRT-PCR analysis at 10 days post-transduction. Gene expression was determined relative to pooled housekeeping genes Glyceraldehyde 3-phosphate dehydrogenase (GAPDH), Beta-actin (B-actin), Ribosomal Protein S23 (RPS23) and Splicing Factor 3a Subunit 1 (SF3A1). (n=2). **b,** Western blot analysis of H2A.Z. g-tubulin was used as the endogenous loading control. Representative blot shown from four biologically independent replicates.

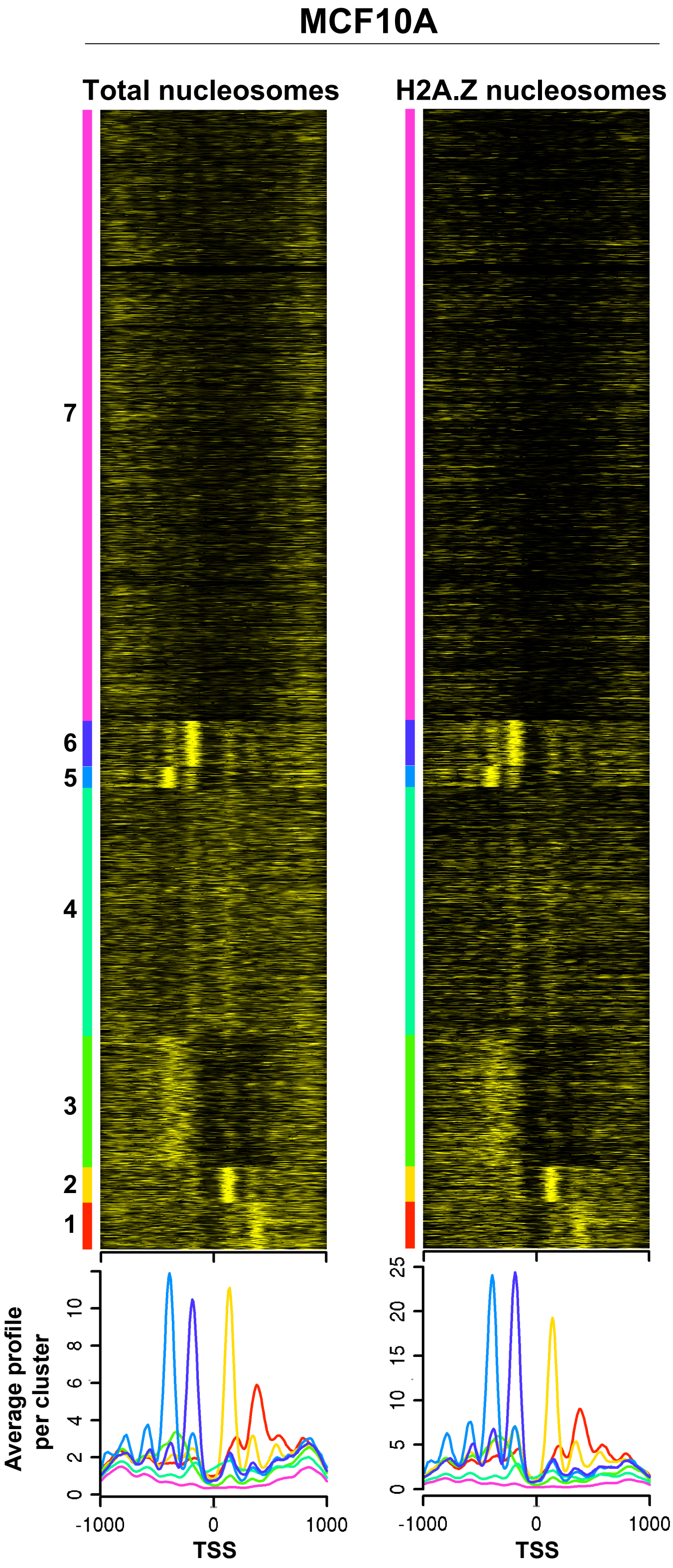

Supplementary Fig. 4. H2A.Z nucleosomes are generally in the most highly occupied total nucleosome positions. Total H2A.Z nucleosomes in MCF10A cells were sorted dependent upon total MTSS-seq nucleosome clustering (as in Figure 1a).

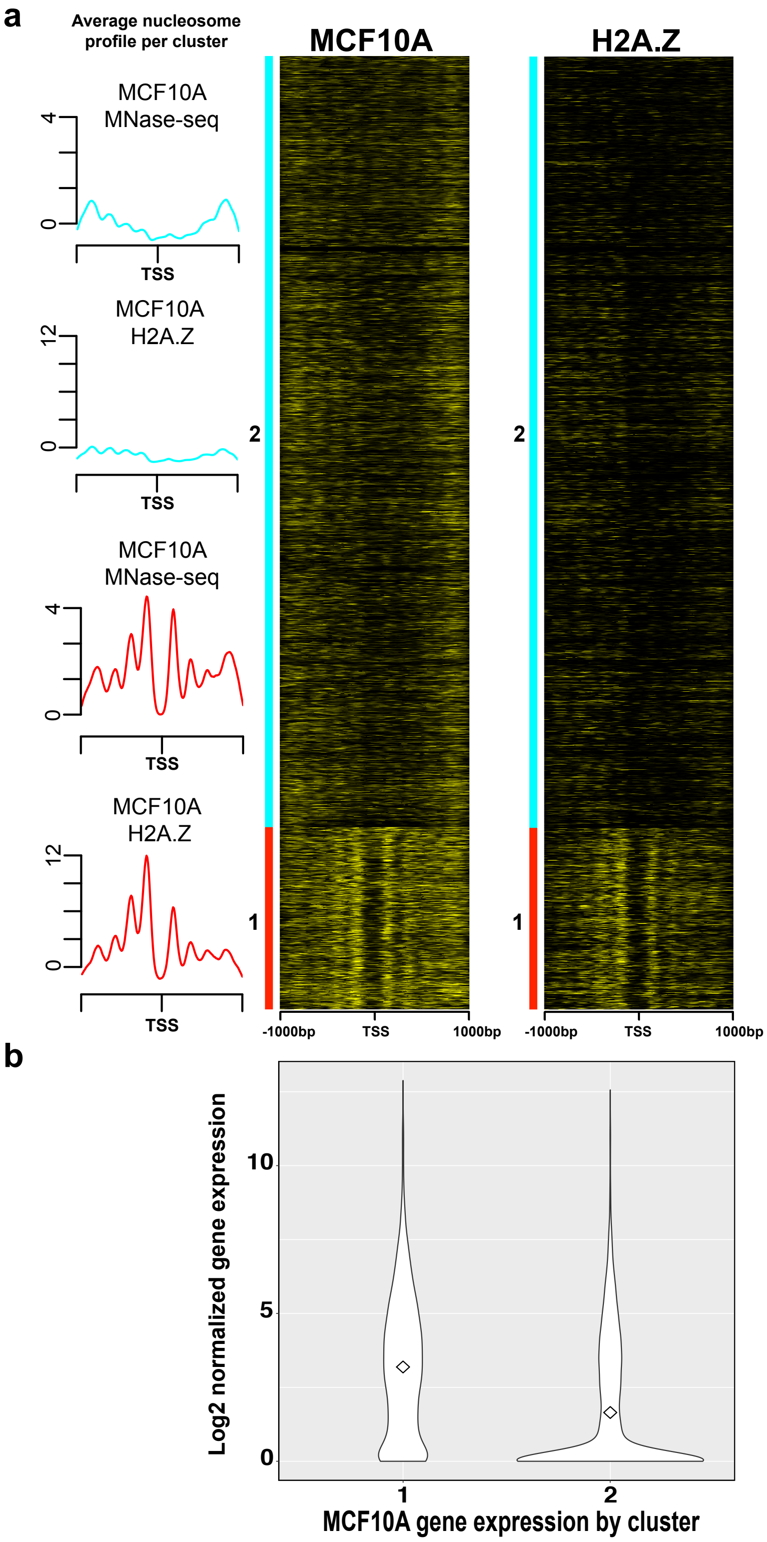

Supplementary Fig. 5. Heatmaps displaying total mTSS-seq and H2A.Z ChIP-Seq data for all human RNA Pol II genes centered on the TSS (+/-1kb). a, K-means clustering where k=2 produces distinct clusters that display high (cluster 1) and low (cluster 2) nucleosome occupancy at the specific -2, -1, +1, and +2 locations relative to the TSS. **b**, Below the heatmaps are gene expression violin plots revealing that high nucleosome occupancy is correlated with higher mean gene expression. RNA-seq was performed in 3 independent biological replicates.

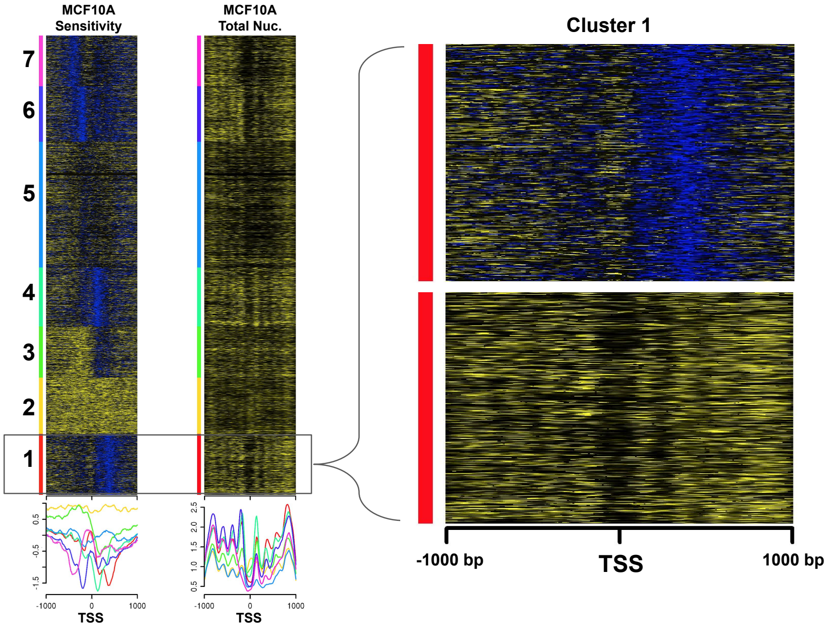

Supplementary Fig. 6. A heatmap of total nucleosomes in MCF-10A cells sorted dependent upon MNase sensitivity clustering. Heatmaps displaying mTSS-seq data for all human promoters centered on the TSS (+/-1kb). Total nucleosome data for MCF-10A was sorted on the same sort order as MCF-10A MNase-sensitivity data. Also shown is a zoomed in image of the +2 MNase-resistant nucleosome cluster.

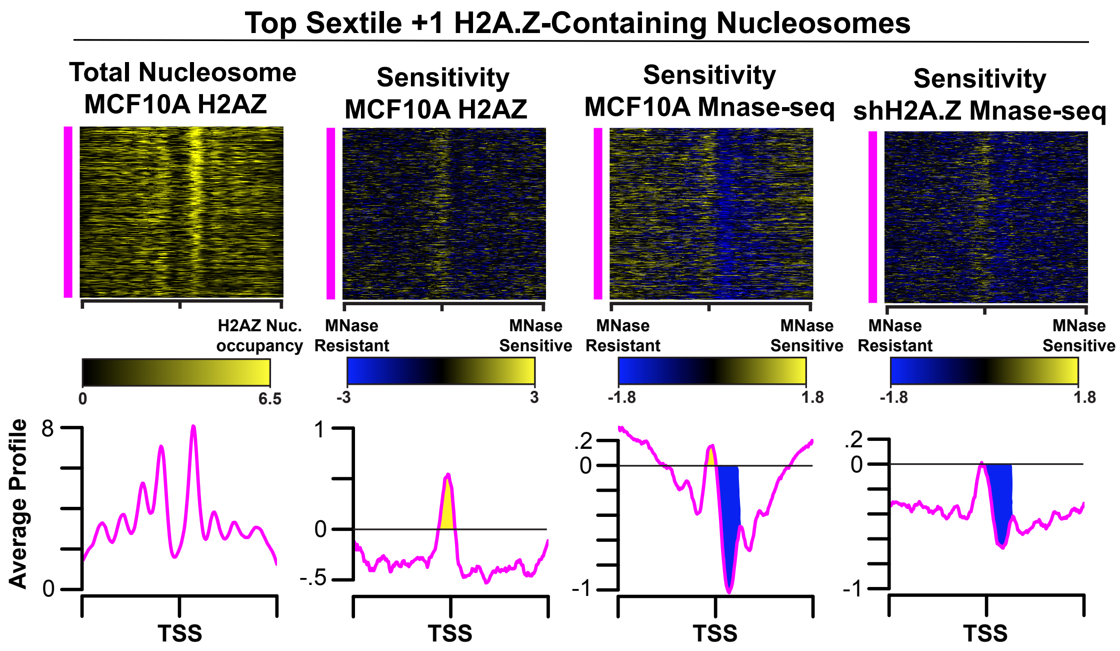

Supplementary Fig. 7. H2A.Z knockdown results in the loss of +1 nucleosome MNase resistance. The top sextile of expressed genes in MCF10A cells were sorted on maximum occupancy of the +1 nucleosome, and the resulting heatmaps for total and H2A.Z-containing nucleosomes and corresponding MNase sensitivity are in the same sort order with average occupancy of the cluster displayed below each heatmap. The yellow peak shows the H2A.Z MSN at the TSS. The blue inverted peak shows the MRN at the +1 position. This resistance is lost following the knockdown of H2A.Z.

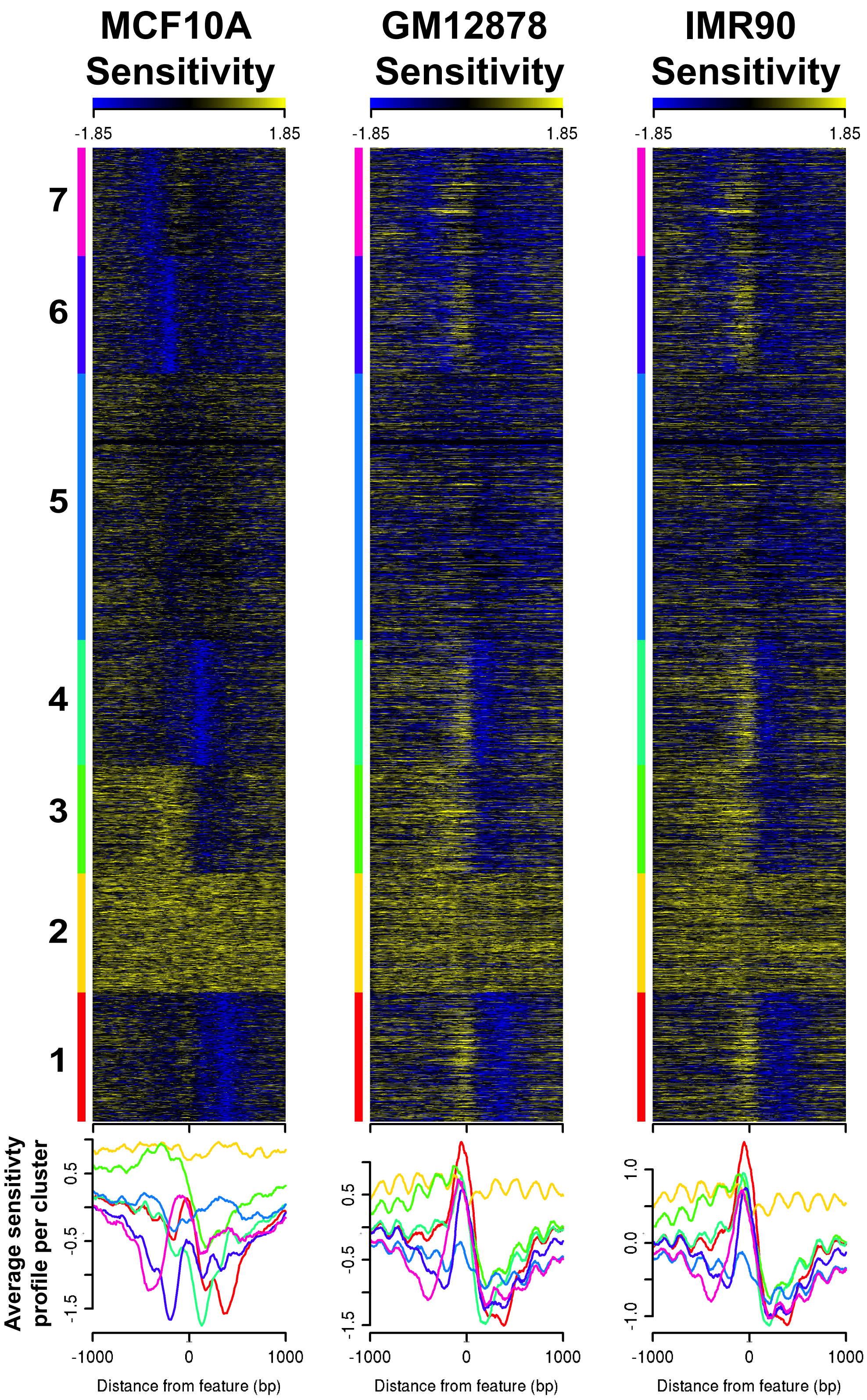

Supplementary Fig. 8. MNase sensitivity data for GM12878 and IMR90 cell lines. Data from these tier 1 and 2 ENCODE cell lines recapitulates similar MSN and MRN clusters observed in MCF10A cells. All human promoters plotted +/- 1000bp surrounding the TSS and sorted via K-means clustering (k=7).

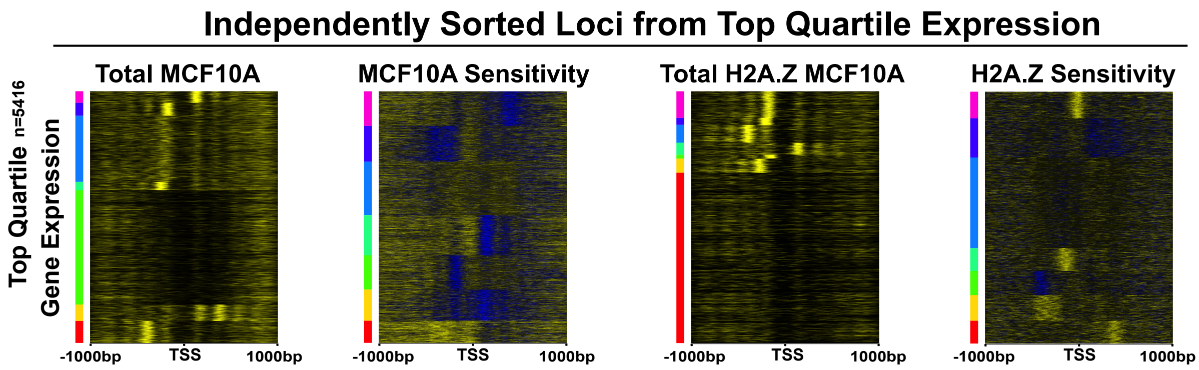

Supplementary Fig. 9. Independently sorted heatmaps of the top quartile of expressed genes in MCF10A cells. Total bulk nucleosomes and H2A.Z-containing nucleosome occupancy, and total and H2A.Z MNase sensitivity profiles for the top quartile of expressed genes in MCF10A cells (Figure 2a) were each sorted independently with K-means clustering (k=7). This reproduces the same types of promoter clusters as observed in Figure 1.

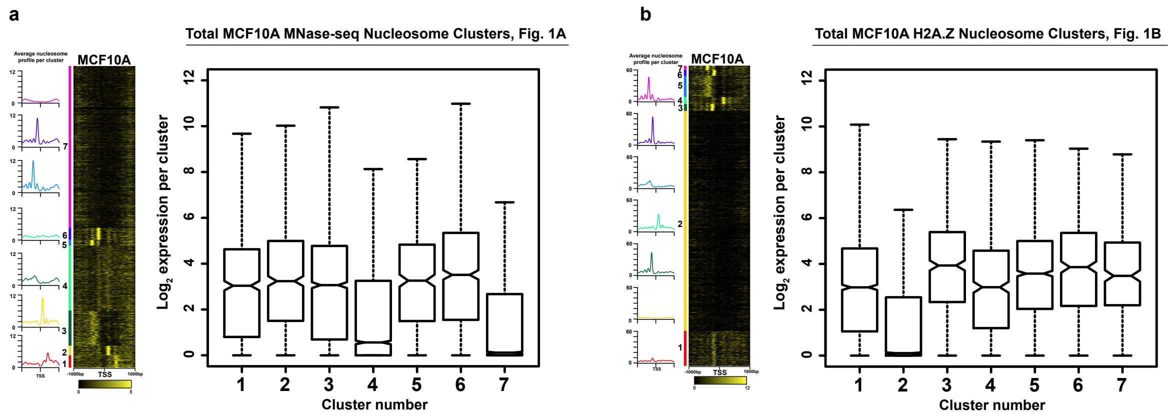

Supplementary Fig. 10. High nucleosome occupancy is correlated with high levels of gene expression. Gene expression box plots for each of the seven **a,** total MNase-seq, and **b,** H2A.Z promoter clusters for MCF-10A cells as shown in Figure 1a and b, respectively. Box plots represent the data in quartiles with the median shown as a notch, with the middle 50% of the data contained in the box, and the minima and maxima shown with the upper and lower whiskers representing Q1 and Q4. RNA-seq was performed in 3 independent biological replicates.

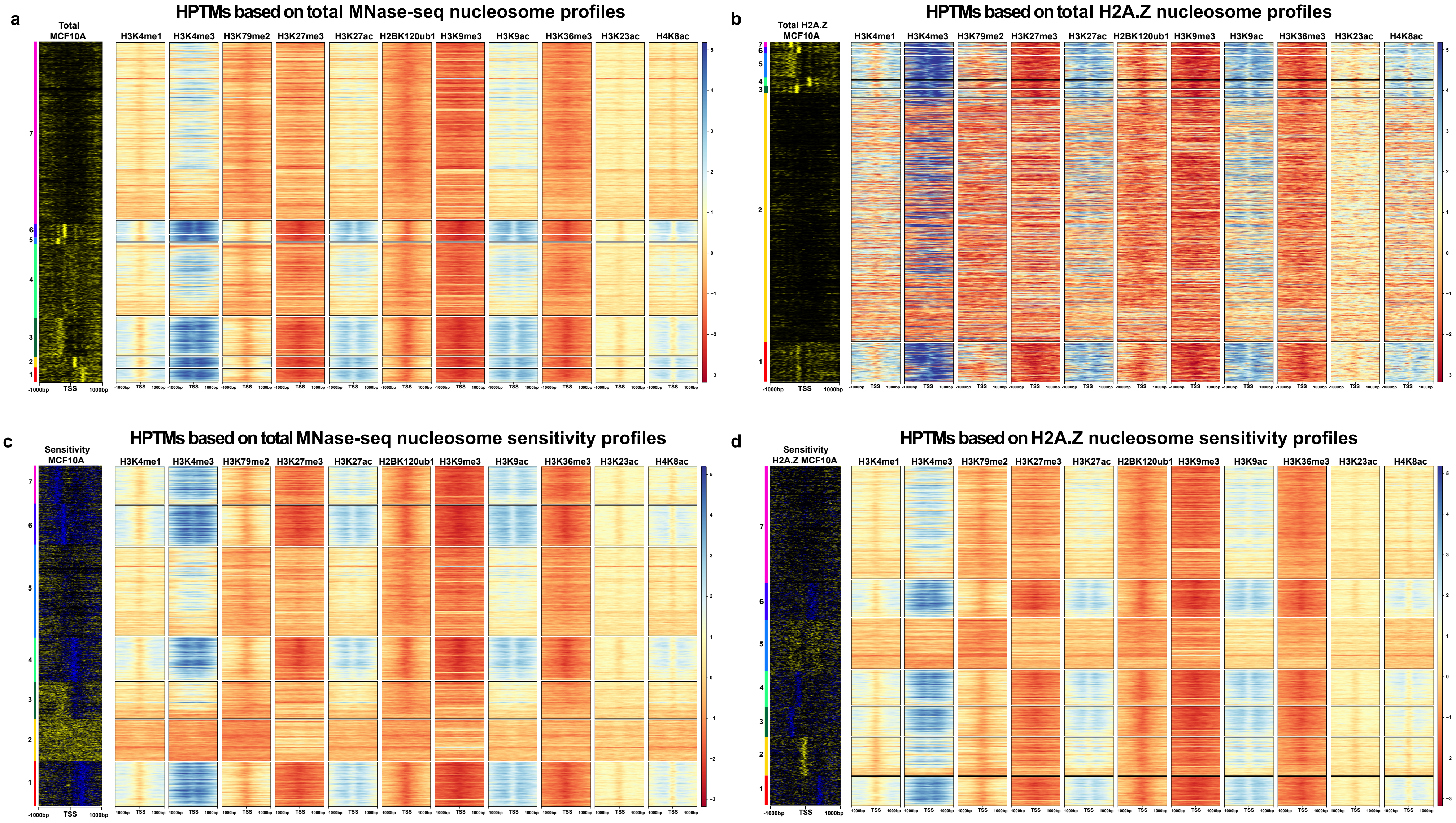

Supplementary Fig. 11. Heatmaps displaying histone posttranslational modifications for different promoter classes based on total mTSS-seq, H2A.Z ChIP-Seq, total and H2A.Z MNase sensitivity data. a, HPTMs based on total mTSS-seq profiles. **b**, HPTMs based on H2A.Z ChIP-seq profiles. **c**, HPTMs based on total mTSS-seq sensitivity profiles. **d**, HPTMs based on H2A.Z mTSS-seq sensitivity profiles.

a MCF10A H2A.Z nucleosomes

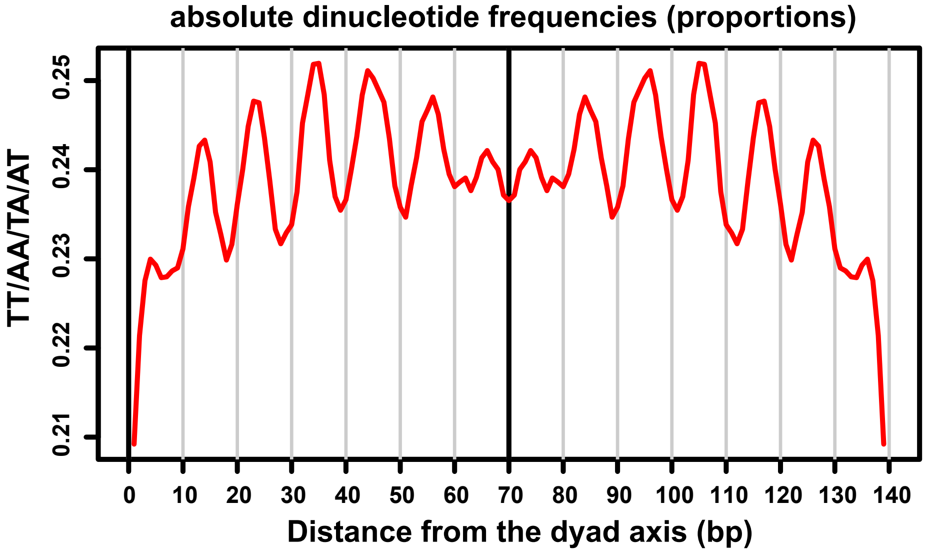

b MCF10A total nucleosomes

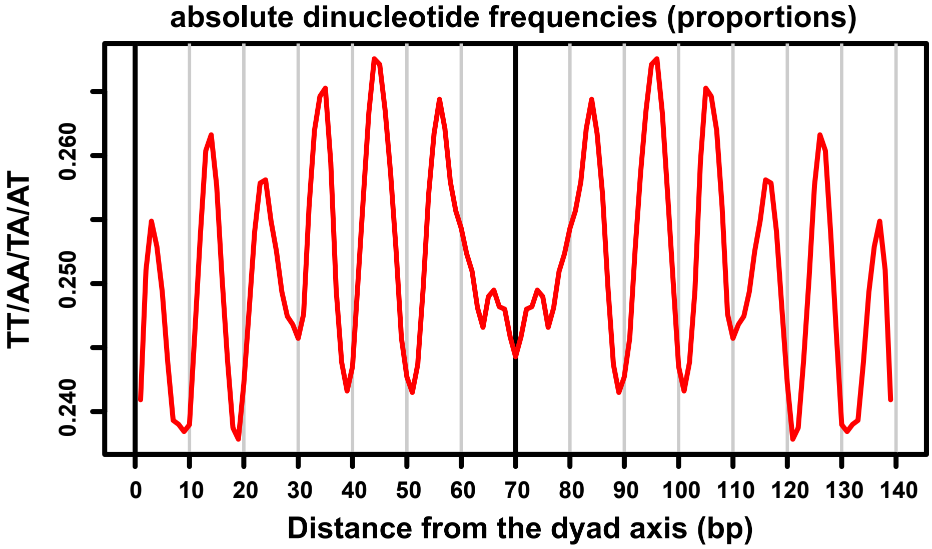

Supplementary Fig. 12. Periodic occurrence of AA/TT/AT/TA dinucleotides. Periodic occurrence was calculated for center aligned nucleosomal-sized fragments (147-150 bp) for **a**) total MCF10A nucleosomes and **b**) H2A.Z-containing MCF10A nucleosomes. The y-axis represents the frequency of AA/TT/AT/TA dinucleotides.

Supplementary Table 1. RT-PCR primer sequences.

GAPDH:

S: 5'- CATCAATGACCCCTTCATTG-3'

AS: 5'- GATCTCGCTCCTGGAAGATG-3'

β -actin:

S: 5'- CCAACCGCGAGAAGATGA-3'

AS: 5'- TCCATCACGATGCCAGTG-3'

RPS23:

S: 5'- CGGTGCTTCTCTCTTTCGCT-3'

AS: 5'- ATGCCACTTCTGGTCTCGT-3'

SF3A1:

S: 5'- AAGGGTCCAGTGTCCATCAAAGT-3'

AS: 5'- GCCATGTTCTAGTAAGCCAGTGAG -3'

H2A.Z

S: 5'-GAAAGGCCAAGACAAAGGC-3'

AS: 5'-ATGGCTGCGCTGTACACAG-3'

Supplementary Table 2. Sequencing data information for H2A.Z ChIP-seq and mTSS-seq experiments.

Samples	reads	aligned concordantly 0 times	aligned concordantly exactly 1 time	% overall alignment rate
A_H2AZ_H_r1	14584997	70393	13302505	99.51%
A_H2AZ_H_r2	15896540	77228	14499357	99.51%
A_H2AZ_L_r1	10827090	69495	9682061	99.36%
A_H2AZ_L_r2	8230126	51441	7355843	99.37%
A_Inp_H_r1	12882694	78004	11210766	99.39%
A_Inp_H_r2	8000794	45651	6968355	99.43%
A_Inp_L_r1	2695517	18258	2194999	99.32%
A_Inp_L_r2	4947519	36277	4023063	99.27%
A_shH2AZ_Inp_H_r1	9339216	43170	8016603	99.54%
A_shH2AZ_Inp_H_r2	7934626	31461	6817671	99.60%
A_shH2AZ_Inp_L_r1	5178937	25668	3831635	99.50%
A_shH2AZ_Inp_L_r2	6893528	31877	5176153	99.54%
A_TGFb_H2AZ_H_r1	29020173	115471	26519106	99.60%
A_TGFb_H2AZ_H_r2	17281968	70911	15788153	99.59%
A_TGFb_H2AZ_L_r1	7806338	49056	7002667	99.37%
A_TGFb_H2AZ_L_r2	6842409	40912	6131049	99.40%
A_TGFb_Inp_H_r1	6963902	42406	6034697	99.39%
A_TGFb_Inp_H_r2	12783775	72594	11088634	99.43%
A_TGFb_Inp_L_r1	3810680	25302	2998650	99.34%
A_TGFb_Inp_L_r2	1854471	10218	1461324	99.45%
CA1a_H2AZ_H_r1	60522981	214306	54956034	99.65%
CA1a_H2AZ_H_r2	33205597	123821	30146501	99.63%
CA1a_H2AZ_L_r1	8400294	45387	7495215	99.46%
CA1a_H2AZ_L_r2	11053303	50242	9868047	99.55%
CA1a_Inp_H_r1	5575937	24348	4774600	99.56%
CA1a_Inp_H_r2	7432509	30724	6362048	99.59%
CA1a_Inp_L_r1	3327773	16164	2634546	99.51%
CA1a_Inp_L_r2	5547533	28568	4408664	99.49%

Supplementary References

1. Gaffney, D.J. et al. Controls of nucleosome positioning in the human genome. *PLoS Genet* **8**, e1003036 (2012).
2. Cole, L., Kurscheid, S., Nekrasov, M., Domaschenz, R., Vera, D.L., Dennis, J.H. and Tremethick, D.J. Multiple roles of H2A.Z in regulating promoter chromatin architecture in human cells. doi.org/10.5281/zenodo.4478453 (2021).
3. Eden, E., Navon, R., Steinfeld, I., Lipson, D. & Yakhini, Z. GOrilla: a tool for discovery and visualization of enriched GO terms in ranked gene lists. *BMC bioinformatics* **10**, 48 (2009).
