# Supplementary material for: Multiple roles of H2A.Z in regulating promoter chromatin architecture in human cells
Source: Nat Commun. 2021 May 5;12:2524. doi: 10.1038/s41467-021-22688-x (PMC8100287; doi:10.1038/s41467-021-22688-x)
Supplement: Supplementary file 3 — Description of Addition Supplementary Files [file 41467_2021_22688_MOESM3_ESM.pdf]

Supplementary Data 1. Results of the differential RNA-Seq gene expression analysis [2].

Supplementary Data 2. Gene ontology analysis of the seven different total nucleosome, and MNase sensitivity promoter clusters in MCF-10A cells. p-values are determined by hypergeometric null model as described in Eden et al. 2009 [3].

Supplementary Data 3. Alluvial Table of promoters that change in chromatin structure and expression following H2A.Z knockdown, TGF-β treatment and malignant transformation [2].

Supplementary Data 4. Table of gene ontology enrichment of promoters that change in chromatin structure and expression based on Supplementary Table 3. p-values are determined by hypergeometric null model as described in Eden et al. 2009 [3].

Supplementary Data 5. 103 transcription factors are expressed that recognize DNA motifs which overlap with sub-nucleosomal DNA peaks in shH2A.Z MCF-10A cells [2].
