# Supplementary material for: Multiple roles of H2A.Z in regulating promoter chromatin architecture in human cells
Source: Nat Commun. 2021 May 5;12:2524. doi: 10.1038/s41467-021-22688-x (PMC8100287; doi:10.1038/s41467-021-22688-x)
Supplement: Supplementary file 9 — Reporting Summary [file 41467_2021_22688_MOESM9_ESM.pdf]

nature research

Corresponding author(s): Jonathan H. Dennis, David J. Tremethick

Last updated by author(s): Feb 1, 2021

Reporting Summary

Nature Research wishes to improve the reproducibility of the work that we publish. This form provides structure for consistency and transparency in reporting. For further information on Nature Research policies, see Authors & Referees and the Editorial Policy Checklist .

Statistics

For all statistical analyses, confirm that the following items are present in the figure legend, table legend, main text, or Methods section.

n/a	Confirmed	
[ ]	[x]	The exact sample size (*n*) for each experimental group/condition, given as a discrete number and unit of measurement
[x]	[ ]	A statement on whether measurements were taken from distinct samples or whether the same sample was measured repeatedly
[ ]	[x]	The statistical test(s) used AND whether they are one- or two-sided<br>*Only common tests should be described solely by name; describe more complex techniques in the Methods section.*
[x]	[ ]	A description of all covariates tested
[x]	[ ]	A description of any assumptions or corrections, such as tests of normality and adjustment for multiple comparisons
[x]	[ ]	A full description of the statistical parameters including central tendency (e.g. means) or other basic estimates (e.g. regression coefficient) AND variation (e.g. standard deviation) or associated estimates of uncertainty (e.g. confidence intervals)
[ ]	[x]	For null hypothesis testing, the test statistic (e.g. *F*, *t*, *r*) with confidence intervals, effect sizes, degrees of freedom and *P* value noted<br>*Give P values as exact values whenever suitable.*
[x]	[ ]	For Bayesian analysis, information on the choice of priors and Markov chain Monte Carlo settings
[x]	[ ]	For hierarchical and complex designs, identification of the appropriate level for tests and full reporting of outcomes
[x]	[ ]	Estimates of effect sizes (e.g. Cohen's *d*, Pearson's *r*), indicating how they were calculated

Our web collection on statistics for biologists contains articles on many of the points above.

Software and code

Policy information about availability of computer code

Data collection	Base-calling was performed using the "bcl2fastq2" script (V2.16) of the CASAVA pipeline.
Data analysis	bcl2fastq2 == v2.16 (https://emea.support.illumina.com/content/dam/illumina-support/documents/documentation/software_documentation/bcl2fastq/bcl2fastq2-v2-20-software-guide-15051736-03.pdf)<br>bowtie2==2.3.4.3 (https://github.com/BenLangmead/bowtie2/releases/tag/v2.3.4.3)<br>samtools==1.9 (https://github.com/samtools/samtools/releases/tag/1.9)<br>macs2==2.1.0 (https://github.com/taoliu/MACS/releases/tag/2015.4.20)<br>idr==2.0.3 (https://github.com/nboley/idr/releases/tag/2.0.3)<br>MEME suite==5.0.5 (http://meme-suite.org/)<br>R==3.5.1 (https://cran.r-project.org/src/base/R-3/R-3.5.1.tar.gz)<br>python==2.7.15/3.6.7 (https://www.python.org/downloads/)<br>travis== v0.1(https://github.com/dvera/travis)<br>genmat==v0.1 (https://github.com/dvera/genmat)<br>gyro== v0.1 (https://github.com/dvera/gyro)<br>alluvial== version: 0.1-2 (https://github.com/mbojan/alluvial)<br>BSgenome== v1.42.0 (https://bioconductor.riken.jp/packages/3.4/bioc/manuals/BSgenome/man/BSgenome.pdf)<br>sra-tools== v2.10.1 (https://github.com/ncbi/sra-tools)<br>deeptools2== (deeptools.ie-freiburg.mpg.de)<br>picard== v2.20.1 (https://broadinstitute.github.io/picard/)

For manuscripts utilizing custom algorithms or software that are central to the research but not yet described in published literature, software must be made available to editors/reviewers. We strongly encourage code deposition in a community repository (e.g. GitHub). See the Nature Research guidelines for submitting code & software for further information.

Data

Policy information about availability of data

All manuscripts must include a data availability statement. This statement should provide the following information, where applicable:

- Accession codes, unique identifiers, or web links for publicly available datasets
- A list of figures that have associated raw data
- A description of any restrictions on data availability

The data discussed in this publication have been deposited in NCBI's Gene Expression Omnibus 64. The parent Super Series containing all data is accessible through GEO Series accession number GSE134299 (https://www.ncbi.nlm.nih.gov/geo/query/acc.cgi?acc=GSE134299), the MNase-seq and ChIP-seq data are available through GEO Series GSE134297 (https://www.ncbi.nlm.nih.gov/geo/query/acc.cgi?acc=GSE134297), and the RNA-seq data is accessible through GEO Series GSE134298 (https://www.ncbi.nlm.nih.gov/geo/query/acc.cgi?acc=GSE134298).

Field-specific reporting

Please select the one below that is the best fit for your research. If you are not sure, read the appropriate sections before making your selection.

[x] Life sciences [ ] Behavioural & social sciences [ ] Ecological, evolutionary & environmental sciences

For a reference copy of the document with all sections, see nature.com/documents/nr-reporting-summary-flat.pdf

Life sciences study design

All studies must disclose on these points even when the disclosure is negative.

Sample size	N/A
Data exclusions	N/A
Replication	The sequencing data for ChIP-seq and MNase-seq was performed in 2 independent biological replicates. RNA-seq was performed in 3 independent biological replicates.
Randomization	N/A
Blinding	N/A

Reporting for specific materials, systems and methods

We require information from authors about some types of materials, experimental systems and methods used in many studies. Here, indicate whether each material, system or method listed is relevant to your study. If you are not sure if a list item applies to your research, read the appropriate section before selecting a response.

Materials & experimental systems

n/a	Involved in the study	
[ ]	[x]	Antibodies
[ ]	[x]	Eukaryotic cell lines
[x]	[ ]	Palaeontology
[x]	[ ]	Animals and other organisms
[x]	[ ]	Human research participants
[x]	[ ]	Clinical data

Methods

n/a	Involved in the study	
[ ]	[x]	ChIP-seq
[x]	[ ]	Flow cytometry
[x]	[ ]	MRI-based neuroimaging

Antibodies

Antibodies used	anti - H2A.Z antibody-made in house and distributed by Millipore/Sigma.
Validation	This antibody has been validated and used in numerous publications e.g. Cell Reports. 21, 943-952 (2017), Plos Genet 13, e1006633 (2017), NSMB 19, 1076-1083 (2012), NSMB 19, 25-30 (2012).

Eukaryotic cell lines

Policy information about cell lines

Cell line source(s)	MCF 10A cells were obtained from the ATCC and MCF10Ca1a were obtained from the Barbara Ann Karmanos Cancer Institute, having a place of business at 4100 John R, Detroit, Michigan 48201 ("KCI").
Authentication	We determined the relative mRNA expression levels of KLK5, NFE2L3, LIF and TNFSF10 using RT-qPCR to characterize each of these cell lines based on results from the following published study.<br><br>RHEE, D. K., PARK, S. H. & JANG, Y. K. 2008. Molecular signatures associated with transformation and progression to breast cancer in the isogenic MCF10 model. Genomics, 92, 419-428
Mycoplasma contamination	All cell lines tested negative for mycoplasma contamination.<br><br>Forward primers<br>◦ Myco-5-1 CGCCTGAGTAGTACGTTCGC<br>◦ Myco-5-2 CGCCTGAGTAGTACGTACGC<br>◦ Myco-5-3 TGCCTGAGTAGTACATTCGC<br>◦ Myco-5-4 TGCCTGGGTAGTACATTCGC<br>◦ Myco-5-5 CGCCTGGGTAGTACATTCGC<br>◦ Myco-5-6 CGCCTGAGTAGTATGCTCGC<br><br>Reverse primers<br>◦ Myco-3-1 GCGGTGTGTACAAGACCCGA<br>◦ Myco-3-2 GCGGTGTGTACAAAACCCGA<br>◦ Myco-3-3 GCGGTGTGTACAAACCCCGA
Commonly misidentified lines<br>(See ICLAC register)	No commonly misidentified cell lines were used.

ChIP-seq

Data deposition

☒ Confirm that both raw and final processed data have been deposited in a public database such as GEO.

☒ Confirm that you have deposited or provided access to graph files (e.g. BED files) for the called peaks.

Data access links<br>*May remain private before publication.*	The data discussed in this publication have been deposited in NCBI's Gene Expression Omnibus (Edgar et al., 2002) and are accessible through GEO Series accession number GSE134299
Files in database submission	A_H2AZ_H_r1_R1<br>A_H2AZ_H_r2_R1<br>A_H2AZ_L_r1_R1<br>A_H2AZ_L_r2_R1<br>A_Inp_H_r1_R1<br>A_Inp_H_r2_R1<br>A_Inp_L_r1_R1<br>A_Inp_L_r2_R1<br>A_shH2AZ_Inp_H_r1_R1<br>A_shH2AZ_Inp_H_r2_R1<br>A_shH2AZ_Inp_L_r1_R1<br>A_shH2AZ_Inp_L_r2_R1<br>A_TGFb_H2AZ_H_r1_R1<br>A_TGFb_H2AZ_H_r2_R1<br>A_TGFb_H2AZ_L_r1_R1<br>A_TGFb_H2AZ_L_r2_R1<br>A_TGFb_Inp_H_r1_R1<br>A_TGFb_Inp_H_r2_R1<br>A_TGFb_Inp_L_r1_R1<br>A_TGFb_Inp_L_r2_R1<br>CA1a_H2AZ_H_r1_R1<br>CA1a_H2AZ_H_r2_R1<br>CA1a_H2AZ_L_r1_R1

CA1a_H2AZ_L_r2_R1
CA1a_Inp_H_r1_R1
CA1a_Inp_H_r2_R1
CA1a_Inp_L_r1_R1
CA1a_Inp_L_r2_R1

Genome browser session (e.g. UCSC)

No longer applicable

Methodology

Replicates

2 biological

Sequencing depth

All experiments were paired-end 50bp reads, with fragment size ~200bp. The average sequencing depth for the experimental conditions was 46 million reads, with an average of 41 million reads which align concordantly exactly 1 time. Sequencing depth table included in supplemental materials.

Antibodies

anti sheep H2A.z (see above).

Peak calling parameters

```
#Peak calling parameters for H2A.Z ChIP-Seq:
macs2 callpeak -g 40999507\ # genome size adjusted to bp covered by sequence capture
                              -t ${dataDir}/A_H2AZ_H_r2_R1.final.bam\
                              -c ${dataDir}/A_Inp_H_r1_R1.final.bam\
                                ${dataDir}/A_Inp_H_r2_R1.final.bam\
                              --qvalue 0.99\
                              --outdir ${outputDir}/A_H2AZ_H_r2_R1\
                              -n A_H2AZ_H_r2_R1\
                              --bdg\
                              --SPMR\
                              --cutoff-analysis\
                              --call-summits\
                              --nomodel\
                              --extsize 147

# followed by irreproducibility of discovery rate (IDR) analysis
idr --samples ${outputDir}/A_H2AZ_H_r1_R1/A_H2AZ_H_r1_R1_peaks.narrowPeak\
                 ${outputDir}/A_H2AZ_H_r2_R1/A_H2AZ_H_r2_R1_peaks.narrowPeak \
     --input-file-type narrowPeak\
     --output-file H2AZ_H_idr_values.txt \
     --log-output-file H2AZ_H_idr_log.txt \
     --soft-idr-threshold 0.1 \
     --plot

#Peak calling parameters for small fragment peaks for MNase and H2A.Z ChIP-Seq:
macs2 callpeak -f BED \
                    -g 40999507 # genome size adjusted to bp covered by sequence capture\
                    -t {input.chip}\
                    -n {params.name}\
                    --nomodel\
                    --extsize 125\
                    --outdir {output.outDir}\
                    --call-summits\
                    -p 0.1\
                    --bdg\
                    --trackline
```

Data quality

Data quality of peak sets was controlled by generating of summary statistics of macs2 and idr results, and visual inspection of a quasi-random selection of peaks. The R script is available at:
https://github.com/JCSMR-Tremethick-Lab/MCF10APromoterAnalysis/blob/master/R/TFBSAnalysis/peakCallingParameterOptimisationForSmallFragments.R

We applied IDR methodology to generate a unified peak set from both biological replicates of the H2A.Z high digest libraries. For these we found 1326 peaks to be reproducible with a global IDR value > 2 and a signal value (fold enrichment) > 5.

Software

bowtie2==2.3.4.3 (https://github.com/BenLangmead/bowtie2/releases/tag/v2.3.4.3)
samtools==1.9 (https://github.com/samtools/samtools/releases/tag/1.9)
macs2==2.1.0 (https://github.com/taoliu/MACS/releases/tag/2015.4.20)
idr==2.0.3 (https://github.com/nboley/idr/releases/tag/2.0.3)
MEME suite==5.0.1 (http://meme-suite.org/)
R==3.5.1 (https://cran.r-project.org/src/base/R-3/R-3.5.1.tar.gz)

python==2.7.15/3.6.7 (https://www.python.org/downloads/)

all custom scripts for peak calling, post processing and small fragments analysis are available at github:
https://github.com/JCSMR-Tremethick-Lab/MCF10APromoterAnalysis/tree/master/R/TFBSAnalysis
https://github.com/JCSMR-Tremethick-Lab/MCF10APromoterAnalysis/blob/master/snakemake/rules/macs2_peak_calling.py
https://github.com/JCSMR-Tremethick-Lab/MCF10APromoterAnalysis/blob/master/snakemake/rules/shortFragments_macs2_peak_calling.py
https://github.com/JCSMR-Tremethick-Lab/MCF10APromoterAnalysis/blob/master/snakemake/rules/shortFragments_meme_processing.py

additional workflows and scripts for the analysis of previously published histone PTM ChIP-Seq can be found here
https://github.com/skurscheid/mcf10_promoter_profiling
